# Effects of Apolipoprotein E Isoforms in Diabetic Nephropathy of Chinese Type 2 Diabetic Patients

**DOI:** 10.1155/2017/3560920

**Published:** 2017-02-23

**Authors:** YongWei Jiang, Liang Ma, ChengWu Han, Qian Liu, Xiao Cong, YaPing Xu, TingTing Zhao, Ping Li, YongTong Cao

**Affiliations:** ^1^Clinical Laboratory, China-Japan Friendship Hospital, Beijing, China; ^2^Graduate School of Peking Union Medical College, Beijing, China; ^3^Beijing Key Lab Immune-Mediated Inflammatory Diseases, Institute of Clinical Medical Science, China-Japan Friendship Hospital, Beijing, China; ^4^Department of Pathophysiology, Institute of Clinical Medical Science, China-Japan Friendship Hospital, Beijing, China

## Abstract

Diabetic nephropathy (DN) is one of the major chronic complications of diabetes. Genetic polymorphism of Apolipoprotein E (ApoE) has been proposed to participating in DN. The purpose of the study was to evaluate the relationship between ApoE genetic polymorphism and the presence of DN in Chinese type 2 diabetic patients. We studied 845 diabetic patients who were divided into DN group (*n* = 429) and control group (*n* = 416). ApoE genotype was determined by ApoE genotyping chip and the plasmatic biochemical characterization was performed on all subjects. There were differences (*P* < 0.001) in HbA1c, creatinine, and urinary albumin between the two groups. The ApoE *ε*2 allelic frequency was 7.69% in DN group versus 3.49% in control group (OR = 2.22, 95% CI = 1.41–3.47, and *P* < 0.05), as expected, ApoE E2/E2 and E2/E3 genotype frequency were higher in DN group (13.75% versus 6.49%, *P* < 0.05). The ApoE *ε*4 allelic frequency was 7.93% in DN group versus 11.54% in control group (OR = 0.70, 95% CI = 0.50–0.97, and *P* < 0.05), and DN group presented a lower frequency of ApoE E3/E4 and E4/E4 genotype frequency (14.91% versus 19.96%, *P* < 0.05). These results suggest ApoE *ε*2 allele may be a risk factor; however ApoE *ε*4 allele may play a protective role of DN in Chinese type 2 diabetic patients.

## 1. Introduction

Diabetic nephropathy (DN) is one of the major chronic complications of diabetes mellitus (DM), and approximately 35 percent of type 2 diabetes mellitus (T2DM) patients eventually developed DN. Accompanied with high prevalence of diabetes, DN has now become the major cause of end-stage renal disease (ESRD). About 50 percent new patients required dialysis treatment in Europe is caused by DN [1]. In China, DN has become the second cause of ESRD only to chronic glomerulonephritis, and from 2010 the leading cause of new-onset ESRD in Beijing, China, has converted from chronic glomerulonephritis to DN [2]. Clinical observations indicate that some T2DM patients under strict glycemic control have emerged with DN; however, some T2DM patients with poor glycemic control do not appear to be DN, suggesting that hyperglycemia is a necessary but not the only factor of DN. Complex genetic background is involved in DN which may be partly attributed to genetic predisposition [3]. Studies have shown that the occurrence of DN has familial aggregation [4–6]; the incidence of DN in T2DM patients whose parents have DN is significantly higher than those whose parents are without DN [7]. Although DN pathogenesis is not yet very clear, epidemiological studies have found a correlation between DN and a variety of lipid metabolism proteins [8–10].

ApoE is a plasma protein including 299 amino acids which plays an important role in lipid and lipoprotein metabolism. In humans, apoE gene located on the chromosome at position 19q13.2 and SNPs at positions 112 (rs429358) and 158 (rs7412) determine three major alleles: *ε*2 (Cys 112, 158 Cys), *ε*3 (Cys 112, 158 Arg), and *ε*4 (Arg 112, 158 Arg). The alleles form six different phenotypes: 3 homozygous (E2/E2, E3/E3, and E4/E4) and 3 heterozygous (E2/E3, E3/E4, and E2/E4). The E3/E3 phenotype has the widest distribution in healthy people, E3/E4 and E2/E3 phenotypes have fewer distribution than E3/E3, and E2/E2, E4/E4, and E2/E4 phenotype have the lowest distribution [11].

ApoE is a high affinity ligand of several hepatic lipoprotein receptors such as low-density lipoprotein receptor (LDLR) and LDL-related protein (LRP) which plays important roles in triglyceride lipoprotein and cholesterol metabolism [12]. ApoE gene polymorphism is one of the important factors affecting body's lipid levels, in particular serum cholesterol levels. The ApoE affinity to LDL receptor and lipoprotein particles was changed because of its different allele. The ApoE *ε*3 allele (also called “wild-type”) has normal function [13]. Compared with the *ε*3 allele, the affinity of ApoE *ε*2 to LDLR and LRP is reduced, resulting in the reduction of LDL-C and the accumulation of decomposed products of triglyceride-rich lipoproteins in plasma, whereas ApoE *ε*4 carriers showed a reduced LDLR affinity and increased LRP affinity which accelerate the metabolism of triglyceride-rich lipoproteins, leading to elevated serum levels of LDL [14]. Recently, studies have pointed out that ApoE gene has become a potential genetic marker of dyslipidemia and DN [15].

The primary aim of this study was to demonstrate whether ApoE gene polymorphism is an important determinant of DN in Chinese T2DM patients. Secondarily, this genetic study might also add more information about a Chinese population beyond traditional risk factors.

## 2. Materials and Methods

### 2.1. Patients

#### 2.1.1. Diabetic Nephropathy Patients (DN Group)

We studied 429 patients with DN in China-Japan Friendship Hospital in Beijing, China, from January 2014 to October 2016. All recruited DN patients fulfilled the following inclusion criteria: (1) patients had been diagnosed with T2DM according to the criteria of World Health Organization (WHO) 1999, (2) 24 h urinary albumin > 300 mg and urinary albumin creatinine ratio > 30 mg/(gCr), and (3) all patients have no kidney disease history previously, and any disease that causes urinary albumin changes was excluded, such as ketosis, lupus nephritis, and urinary tract infections.

#### 2.1.2. Type 2 Diabetic Patients (Control Group)

We studied 416 T2DM patients in China-Japan Friendship Hospital in Beijing, China, from January 2014 to October 2016. T2DM was diagnosed according to the criteria of World Health Organization (WHO) 1999. All selected patients have diabetic duration > 10 years, negative urinary albumin measured on three different occasions, and urinary albumin creatinine ratio ≤ 30 mg/(gCr).

The protocol was approved by institutional ethical committee of China-Japan Friendship Hospital. Informed consent was obtained from the patients before the study began.

### 2.2. General Survey

A full general revision of antecedents including arterial hypertension and nutritional state was carried out. Plasmatic biochemical characterization included the following: (1) glycemia and lipid profile (total cholesterol, LDL cholesterol, HDL cholesterol, and triglyceride) and renal function (creatinine and urinary albumin) measured by automatic biochemistry analyzer (SIEMENS) and (2) venous blood samples (10 ml) collected from patients after a 48 h low fat diet and a 12 h overnight fast.

### 2.3. Apolipoprotein E Genotype

Genomic DNA was extracted from venous blood according to the manufacturer's recommendations (Qiagen). The concentration of DNA was determined using a NanoDrop 1000 spectrophotometer (Thermo Fisher). DNA samples were frozen at −20°C until processed. For the detection of the three ApoE common alleles (*ε*2, *ε*3, and *ε*4), a commercial ApoE genotyping chip (Sinochips, China) was performed according to the protocols provided by the manufacture. Polymorphic alleles were identified by the fluorescence intensity of the hybridization sites.

### 2.4. Statistical Analysis

Quantitative clinical data (age, BMI, blood pressure, duration of diabetes, triglyceride, total cholesterol, and HDL-C, LDL-C, HbA1c, creatinine, creatinine clearance, and urinary albumin) were non-Gaussian distribution and presented as median (interquartile range), and Wilcoxon test was used to test the differentiation between DN group and control group. Frequencies and percentages described the nonparametric variables and the comparison between these groups was based on the Chi-square test. A logistic regression analysis was carried out using DN as dependent variable. Power calculation was performed by Quanto software version 1.2.4 (http://biostats.usc.edu/Quanto.html). Data was analyzed with the SPSS 17.0. A *P* value of <0.05 was considered to be statistically significant.

## 3. Results

### 3.1. Clinical and Laboratory Characteristics

The study involved 845 subjects that included 429 type 2 DN patients (257 males and 172 females) as DN group and 416 T2DM patients (229 males and 187 females) as control group (Table 1). Clinical and laboratory characteristics of the two groups are shown in Table 1. There were no significant difference of age, sex, body mass index (BMI), duration of diabetes, and diastolic blood pressure between the two groups. The DN group has the similar triglyceride, total cholesterol, HDL cholesterol, and LDL cholesterol levels with the control group, which may be due to the lipid-lowering therapy for patients with hyperlipidemia and statin lipid-lowering drugs are used extensively. As expected, DN group had higher HbA1c, creatinine, urinary albumin, and lower creatinine clearance levels than control group.

### 3.2. Genotypes and Allelic Frequency of ApoE

We observed differences in the distribution of ApoE genotype between the two groups (Table 2). The E2/E2 and E2/E3 genotype frequency in DN group were higher (Chi^2^, *P* < 0.05) while E3/E4 and E4/E4 genotype frequency were lower (Chi^2^, *P* < 0.05) in DN group than in control group. We also observed differences in the distribution of *ε*2 and *ε*4 alleles of ApoE between both groups (Table 2). The frequency of Apo *ε*2 allele was significantly higher (7.69% versus 3.49%, *P* < 0.05) and the *ε*4 allele (7.93% versus 11.54%, *P* < 0.05) was lower in DN group than in control group. In the allele statistical processing, the APOE E2/E4 patients were excluded.

### 3.3. Association of ApoE Polymorphism with DN Risk

To confirm the association between the ApoE genotyping and the risk of DN occurrence, further multivariate logistic regression analysis was performed. The result shows the risk of getting DN of SNPs rs7412 (ApoE *ε*2) and rs429358 (ApoE *ε*4) (OR and 95% CI values are shown in Table 3). ApoE *ε*2 genotyping could significantly increase the risk of DN comparing with *ε*3 genotyping (OR = 2.22, 95% CI = 1.41–3.47, and *P* < 0.05), while ApoE *ε*4 genotyping may decrease the risk of DN comparing with *ε*3 genotyping (OR = 0.70, 95% CI = 0.50–0.97, and *P* < 0.05). The high value of 95% CI is nearly 1 and may be due to the relatively small sample size which required further research on the expanding sample size.

## 4. Discussion

Recently, ApoE gene polymorphism has been claimed to play a role in DN development. However, a large number of studies into this issue have failed to reach a definitive conclusion. Most studies on the correlation between ApoE *ε*2 allele and T2DN showed that ApoE *ε*2 may be a risk factor of DN, and the *ε*2 allele frequency was significantly higher in DN patients than in T2DM patients [16, 17]. Studies from Korea, Japan, and other countries found that ApoE *ε*2 allele frequency was higher in the macroalbuminuric group than in normoalbuminuric group, which may be associated to clinic albuminuric development in T2DM patients [14, 18]. Several meta-analyses studied the effect of ApoE gene polymorphism and DN concluded that ApoE *ε*2 allele polymorphism correlated with DN occurrence significantly, and ApoE *ε*2 carriers have higher risk of DN occurrence than noncarriers [19–23]. Consistently, our research found that the frequency of ApoE genotype E2/E2 and APOE E2/E3 in DN group were significantly higher than control group in Chinese T2DM patients and, compared with ApoE *ε*3 allele carriers, the occurrence of DN in ApoE *ε*2 allele carriers is about 2.2-fold higher.

However, studies from France and Turkey obtained different results in diabetic patients. They showed that there were no differences in the ApoE genotype distribution between the diabetic group and the healthy group. Furthermore, they found that the incidence of DN in ApoE *ε*2 allele carriers was significantly lower than in the *ε*3 and *ε*4 carriers. Based on the above results, they speculated that LDL cholesterol may play a main role in nephropathy development and they proposed that ApoE *ε*2 reduced the risk of developing nephropathy because of the lower plasma level of total and LDL cholesterol [24–26]. We did not find the difference of total and LDL cholesterol levels between ApoE *ε*2, *ε*3, and *ε*4 carriers in both groups which was consistent with the results of Erdogan [25]. It is clear that the alleles of ApoE are associated with lipid abnormalities; however, in our study, about 2/3 patients in both two groups were diagnosed with hyperlipidemia and most of them has been treated with statins lipid-lowering drugs, which may affect statistics results.

Most of the research showed that ApoE *ε*4 is a protective factor of DN. Horita et al. reported that lack of ApoE *ε*4 allele is a risk factor for diabetic renal failure [27]; Araki reported that ApoE *ε*3 allele frequency was positively correlated with DN [28]. Kimura et al. studied the relationship between T2DN and ApoE genotype and found that the ApoE *ε*4 allele frequency was significantly higher in stable renal function group (17.1%) than in the microalbuminuria group (8.9%) (*P* = 0.03); however, ApoE *ε*3 allele frequency was significantly higher in proteinuria group than in stable renal function group [29]. Several meta-analyses showed that ApoE *ε*4 allele may be a protective factor for DN which can reduce the DN occurrence in T2DM [20, 30]. In our research, the frequency of ApoE *ε*4 allele was significantly different between the DN group and the control group after excluding the patients who have E2/E4 genotype. Simultaneously, the E3/E4 and E4/E4 genotype distribution between DN group (13.98%, 7.93%) and control group (16.83%, 11.54%) was notably different (*P* < 0.05) which was consistent with the above other results.

On the other hand, another meta-analyses have found no correlation between ApoE *ε*4 and progression of DN (OR = 0.93, 95% CI = 0.78–1.11) [31]. A small amount of literature showed that ApoE *ε*4 allele is a risk factor of DN and the effect of lipid increasing of ApoE *ε*4 may accelerate the progression of DN [19]. Yin et al. have recently published a meta-analysis of 29 studies about the correlation of ApoE polymorphism and T2DM in Chinese Han population. They do not only find that ApoE *ε*2 and ApoE *ε*4 alleles are both risk factors for the development of T2DM but also find that ApoE *ε*2 and ApoE *ε*4 alleles are associated with an increased risk of DN. Our results conflicted with their conclusion that ApoE *ε*4 allele is a risk factor of DN. In most of the studies summarized in this meta-analysis, the duration time of DM is short that many potential DN patients may not yet occur DN in the control group, which may lead to statistics confliction. Relatively small sample size summarized by the above meta-analysis may be another reason that leads to difference conclusion which suggests that further large sample studies are needed [23].

There were controversial results in the correlation of ApoE gene polymorphism and DN. The main reason may be because (1) there were racial differences in the distribution of ApoE alleles, (2) different methods used to detect ApoE genotype may affect the accuracy of the results [31], (3) there are bias and confusion bias in patients selection and statistical processing such as small sample size and short duration of diabetes, (4) observation period was too short in some prospective study to observe the DN occurrence; some patients in the control group may have DN some years later, and (5) different studies had different DN diagnosed standards.

Many experimental data and clinical studies have shown that there are relevance between dyslipidemia and glomerular sclerosis. More and more research suggests that hyperlipidemia is involved in diabetic glomerulosclerosis which is a risk factor for the occurrence and development of DN. Currently, the role of ApoE in lipid transport and its following affection on renal function may be the main explanation of the correlation between ApoE polymorphism and DN. In mice with mild renal impairment, ApoE gene knockout can result in a significant increase of plaque size and its aggression in foam cell-rich soft plaque [12]. ApoE *ε*2 mainly through lipid metabolism pathways is involved in DN. Compared with ApoE *ε*3, affinity of ApoE *ε*2 with LDL receptor and LDL particles is reduced, resulting in the accumulation of the decomposition products of triglyceride-rich lipoproteins in plasma.

It has been revealed that there is high positive correlation between ApoE *ε*4 allele and LDL cholesterol [32]; then, how does ApoE *ε*4 allele play a protective role in the development and progression of DN? The mechanism is not clear now, and there may be the following reasons. First, high cholesterol can accelerate the deterioration of renal function in patients with DN; however, the effects of ApoE *ε*4 allele promoting lipid increasing may be significantly weakened in DN patients [31]. However, this mechanism still needs further research support. Second, ApoE *ε*4 allele protective effects on DN might benefit from unrelated features of lipoprotein metabolism. ApoE gene mainly expressed in mesangial cells and then secreted into the extracellular matrix. Because ApoE have a high affinity with extracellular glycosaminoglycans, when ApoE is synthesized, there may appear biological activity changes or/and replacements of growth factors in the partial of extracellular matrix. Kimura et al. reported that the ApoE *ε*4 allele is a factor that reduces the relative risk for DN progression because ApoE is synthesized in the kidney and probably could displace growth factors involved in pathogenic through its junction to glycosaminoglycans [29]. Tumor growth factor-*β* (TGF-*β*) and platelet-derived growth factor (PDGF) play an important role in the pathogenesis of DN which confined to extracellular proteoglycans, suggesting that ApoE may regulate the levels and biological activity of TGF-*β* and PDGF in the extracellular matrix and thereby affecting renal effects of DN. A third possibility is that ApoE *ε*4 gene loci may be associated with a protective gene of kidney and may be a sign of renal protective alleles. Chen et al. studied the ApoE role in the kidney function of rats, finding that rats deficient in ApoE had a proliferation of mesangial cells and overproduction of mesangial matrix which are important aspects that influence the development of kidney diseases including DN, suggesting a protective role for ApoE in kidney function [33]. Moreover, they found that ApoE increased the level of heparan sulfate proteoglycans (HSPG) that inhibit mesangial cell proliferation. Therefore, it can be suggested that the interaction of ApoE with HSPG has a main role in the development of DN and the ApoE *ε*4 allele has the highest regulation capacity of mesangial cell proliferation and mesangial matrix expansion due to an increased interaction with matrix proteoglycans. However, more information is required to find an explanation for the possible protector role of ApoE *ε*4 allele in the development of DN.

In summary, we observed an association between the ApoE polymorphism and DN in Chinese type 2 diabetes patients. Compared with ApoE *ε*3 allele, ApoE *ε*2 allele carriers have a higher proportion of DN, suggesting that ApoE *ε*2 allele may be a risk factor of DN. Individuals carrying ApoE *ε*4 allele showed a lower prevalence of DN which suggest that ApoE *ε*4 allele may be a protective factor of DN. However, there are still limitations which must be clearly acknowledged. Firstly, this study is limited in a relatively small sample size; another is that the genetic association found in this study may not be generalized to other ethnic groups.

## Figures and Tables

**Table 1:** Clinical and laboratory characteristics of patients in DN group and control group.

	DN group (*n* = 429)	Control group (*n* = 416)	*P* value
Age (years)	62.00 (54.00, 68.00)	62.00 (55.00, 69.50)	ns
Sex (male%)	59.91 (257/429)	55.05 (229/416)	ns
BMI (kg/m^2^)	26.25 (24.47, 28.55)	25.40 (23.63, 27.73)	ns
Duration of diabetes (years)	15.00 (9.00, 20.00)	13.00 (11.00, 18.00)	ns
Systolic blood pressure (mmHg)	138.00 (125.00, 150.00)	130.00 (120.00, 140.00)	ns
Diastolic blood pressure (mmHg)	80.00 (73.25, 84.75)	80.00 (73.50, 81.00)	ns
Triglyceride (mmol/L)	1.60 (1.28, 1.96)	1.40 (0.85, 2.03)	ns
Total cholesterol (mmol/L)	4.00 (3.29, 5.13)	3.99 (3.42, 4.67)	ns
HDL cholesterol (mmol/L)	0.92 (0.77, 1.14)	1.02 (0.87, 1.21)	ns
LDL cholesterol (mmol/L)	2.21 (1.74, 2.93)	2.25 (1.82, 2.86)	ns
HbA1c (%)	7.95 (6.50, 9.63)	10.95 (8.08, 17.00)	<0.05
Creatinine (mmol/L)	107.30 (76.15, 187.45)	63.30 (52.95, 75.50)	<0.05
Creatinine clearance (ml/min/1.73 m^2^)	85.50 (58.85, 120.26)	112.80 (93.06, 140.22)	<0.05
Urinary albumin (mg/day)	330.62 (226.33, 438.92)	13.70 (5.71, 28.17)	<0.05

**Table 2:** Genotypes and allelic frequency of ApoE in DN group and control group.

	Genotype					Allele			
	E2/E2^*∗*^	E2/E3^*∗*^	E3/E3	E2/E4	E3/E4^*∗*^	E4/E4^*∗*^	*ε*2^#^	*ε*3	*ε*4^#^
DN group (*n* = 429)	7	52	298	8	60	4	66	708	68
%	1.63	12.12	69.46	1.86	13.98	0.93	7.69	82.52	7.93
Control group (*n* = 416)	2	25	302	4	70	13	29	699	96
%	0.48	6.01	72.60	0.96	16.83	3.13	3.49	84.01	11.54

^*∗*^Genotypes frequency, Chi^2^, *P* < 0.05.

^#^Allelic frequency: *ε*2 (Chi^2^, *P* < 0.05), *ε*4 (Chi^2^, *P* < 0.05).

**Table 3:** Odds ratios and 95% confidence interval for DN under three genetic models.

Genetic models	*P* value	OR (95% CI)
*ε*2 versus *ε*3	<0.05	2.22 (1.41–3.47)
*ε*4 versus *ε*3	<0.05	0.70 (0.50–0.97)
